# Molecular Review of Suspected Alport Syndrome Patients—A Single-Centre Experience

**DOI:** 10.3390/genes16020196

**Published:** 2025-02-04

**Authors:** Paulina Halat-Wolska, Elżbieta Ciara, Michał Pac, Łukasz Obrycki, Dorota Wicher, Katarzyna Iwanicka-Pronicka, Ewelina Bielska, Beata Chałupczyńska, Dorota Siestrzykowska, Grażyna Kostrzewa, Piotr Stawiński, Rafał Płoski, Mieczysław Litwin, Krystyna Chrzanowska

**Affiliations:** 1 Department of Medical Genetics, The Children’s Memorial Health Institute, 04-730 Warsaw, Poland; p.halat@ipczd.pl (P.H.-W.); d.wicher@ipczd.pl (D.W.); k.iwanicka-pronicka@ipczd.pl (K.I.-P.); e.bielska@ipczd.pl (E.B.); b.chalupczynska@ipczd.pl (B.C.); d.siestrzykowska@ipczd.pl (D.S.); p.stawinski@ipczd.pl (P.S.); k.chrzanowska@ipczd.pl (K.C.); 2 Department of Nephrology, Kidney Transplantation and Hypertension, The Children’s Memorial Health Institute, 04-730 Warsaw, Poland; michal.pac@ipczd.pl (M.P.); l.obrycki@ipczd.pl (Ł.O.); m.litwin@ipczd.pl (M.L.); 3 Department of Medical Genetics, Warsaw Medical University, 02-106 Warsaw, Poland; grazyna.kostrzewa@wum.edu.pl (G.K.); rafal.ploski@wum.edu.pl (R.P.)

**Keywords:** Alport syndrome, *COL4A3*, *COL4A4*, *COL4A5*, novel molecular variants, next-generation sequencing, genetic testing

## Abstract

**Background:** Alport syndrome (AS) is a clinically and genetically heterogeneous glomerulopathy resulting from pathogenic variants in *COL4A3, COL4A4,* and *COL4A5*. Genetic diagnosis is increasingly being conducted using next-generation sequencing (NGS). **Methods:** Within eight years, we examined a group of 247 Polish individuals and found in total 138 unrelated probands suspected with AS based on clinical course, laboratory findings, and/or family history, as well as the total of 109 family members. We applied a targeted NGS panel to identify the genetic spectrum of AS. Known and novel variants were revealed, and detailed evaluation was performed according to ACMG/AMP guidelines to classify them as pathogenic/likely pathogenic/VUS changes. Identified genotypes were compared with clinical manifestations: hematuria, proteinuria, chronic kidney disease, sensorineural hearing impairment, ocular abnormalities, and hypertension. **Results:** The molecular background was established in 109/138 probands. Overall, 79 different *COL4A3-COL4A5* changes (56 known and 23 novel) were revealed. About 97% were SNVs, and only two *COL4A5* CNVs were identified. In total, 11 recurrent *COL4A3-COL4A5* variants were observed, including the most frequent *COL4A5*:p.Gly624Asp, accounting for 31% of X-linked AS. **Conclusions:** The use of NGS panel has shown considerable promise in the field of AS, increasing diagnostic rate to 79% and reducing time to diagnosis. The phenotype-driven gene panel, specific for genetic diseases in the pediatric population, is an affordable alternative to WGS and WES, offering comparable diagnostic efficacy and supporting its implementation as a first-line genetic test in rare diseases, including AS. Based on the obtained genotype–phenotype correlation, we assessed that NGS allows us to avoid invasive renal biopsy in AS diagnosis. It provides AS confirmation/exclusion, atypical AS identification, symptomatic/asymptomatic monoallelic *COL4A3-COL4A5* carrier (especially *COL4A5* females) determination, and inheritance pattern establishment. AS diagnosis confirmation enables clinical course prediction and is crucial for the early introduction of renoprotective treatment with renin–angiotensin–aldosterone system blockade, aimed at slowing the disease progression and estimating the risk in family members, which is important for genetic counselling.

## 1. Introduction

Alport syndrome (AS) is a hereditary kidney disease that affects the glomeruli and may be associated with extrarenal manifestations, including sensorineural hearing impairment, ocular abnormalities, and hypertension.

Although classified as a ‘rare disease,’ Alport syndrome is the most common inherited kidney disease and the second leading cause of end-stage renal disease after autosomal dominant polycystic kidney disease. The global prevalence of Alport syndrome is likely underestimated and varies from 1 in 5000 to 1 in 53,000, depending on the studied population [1]. Alport syndrome occurs in 0.5% of newly developed end-stage renal disease in adults and 12.9% in children [2,3].

Pathogenic variants in the *COL4A3*, *COL4A4*, and *COL4A5* genes, which encode collagen IV *α*3, *α*4, and *α*5 chains, underlie the disease and are called the ‘Alport genes’. The *COL4A5* gene resides on the X chromosome and causes X-linked Alport syndrome, while the *COL4A3* and *COL4A4* genes are both located on chromosome 2, resulting in an autosomal dominant or recessive mode of inheritance. The X-linked form is the most common, accounting for approximately 60% of Alport syndrome, while the dominant and recessive forms contribute about 25% and 15%, respectively. Alport syndrome may also follow a rare digenic inheritance pattern, involving changes in two of the three different collagen IV genes noted above [4].

Alport syndrome can manifest across a wide age range, from childhood to late adulthood; however, it most commonly presents during childhood or adolescence. The term ’Alport syndrome’ now encompasses conditions with a variable set of manifestations, ranging from mild to severe kidney impairment, with hematuria as the main symptom. Progressive loss of kidney function may result in chronic kidney disease (CKD) and end-stage renal disease (ESRD), depending on the mode of inheritance. Biallelic *COL4A3* and *COL4A4* variants, as well as hemizygous *COL4A5* changes, are correlated with the most severe phenotype, characterized by progressive CKD, early-onset ESRD, and extrarenal manifestations. Females with heterozygous *COL4A5* variants are not only asymptomatic carriers of X-linked Alport syndrome but also face an increased risk of CKD, early- or late-onset ESRD, and extrarenal manifestations. Similarly, individuals with monoallelic *COL4A3* or *COL4A4* changes are also at risk of progressive CKD and late-onset ESRD, especially if they exhibit hematuria and proteinuria. Additionally, they may present with variable hearing and ocular abnormalities. However, some carriers remain asymptomatic or may have only isolated hematuria. Thus, under the commonly used term ‘autosomal dominant Alport syndrome’ now fall conditions such as benign familial hematuria, thin basement membrane nephropathy, and the carrier state for autosomal recessive Alport syndrome. Recent studies suggest that predicted pathogenic *COL4A5* variants occur in at least 0.04% of the population, and nearly 1% may be at risk of carrying a heterozygous predicted pathogenic *COL4A3* or *COL4A4* variant but are often unrecognized [1,5,6]. The *COL4A5*:c.1871G>A variant is predominant in Central and Eastern Europe and is the most frequent variant in the Polish population, accounting for 39% (44/113) of genetically confirmed X-linked Alport syndrome cases [7]. In a digenic mode of inheritance, clinical features may be more severe than those observed with a monoallelic *COL4A3* or *COL4A4* variant [8].

To date, approximately 3000 pathogenic and likely pathogenic changes, along with 1800 variants of uncertain significance (VUS) in the *COL4A3*, *COL4A4*, and *COL4A5* genes, have been classified in VarSome (one of the largest human genomic variant search engines, https://sso.varsome.com/). Almost twice as many pathogenic and likely pathogenic changes are identified in the *COL4A5* gene as compared to the *COL4A3* or *COL4A4* genes.

Each collagen IV *α* chain, encoded by the ‘Alport genes’, consists of amino- and carboxy-terminal non-collagenous domains, as well as an intermediate collagenous domain containing multiple non-collagenous interruptions within the Gly-Xaa-Yaa repeats. Numerous studies indicate that the location of a pathogenic variant within the collagen α chain domains, along with the variant type (missense, nonsense, frameshift, splice site, gross deletion, or rearrangement), are major determinants of variant severity. Additionally, variants in the amino-terminal region (exons 1–20) are sometimes associated with milder phenotypes, whereas changes in the carboxy-terminal region (exon 21 and beyond) tend to correlate with more severe clinical outcomes due to the structural and functional organization of the collagen IV α chains [9,10].

Nearly half of variants found in the *COL4A3*, *COL4A4*, and *COL4A5* genes are missense changes, generally associated with a milder phenotype. However, further determinants of severity for missense variants include glycine (Gly) versus non-Gly substitutions. Glycine is the smallest amino acid, and replacing it with any larger amino acid disrupts the triple helix formation. The degree of instability caused by the residue replacing Gly with Ala, Ser, or Cys is considered mildly destabilizing, whereas replacing with Arg, Asp, Glu, Trp, or Val is considered highly destabilizing. Therefore, position 1 Gly in the Gly-Xaa-Yaa repeats of the intermediate collagenous domain is generally considered a pathogenic functional domain. Gly residues in the amino- and carboxy-terminal non-collagenous domains, the non-collagenous interruptions in the intermediate collagenous domain, and positions 2 and 3 in the Gly-Xaa-Yaa repeats are not considered critical [10,11].

The remaining variants are nonsense, frameshift, canonical splice site alterations, and gross deletions or rearrangements, which are likely to be pathogenic because loss of function (LOF) is a recognized disease mechanism for these genes, especially for the *COL4A3* and *COL4A4* genes. Loss-of-function variants are also most frequently associated with a more severe phenotype. The missense and loss of function Z-Scores and observed-over-expected (o/e) ratios for missense and LOF variants for collagen IV genes indicate intolerance to this type of variant: *COL4A3* Z-Score missense = 1.995 (o/e = 0.816); LOF = 5.446 (o/e = 0.398); *COL4A4* Z-Score missense = 1.015 (o/e = 0.908); LOF = 4.487 (o/e = 0.473); and *COL4A5* Z-Score missense = 2.499 (o/e = 0.725); LOF = 6.587 (o/e = 0.097). Additionally, *COL4A3* and *COL4A4* genes are likely to be associated with a recessive disease, as the probability of being intolerant to homozygous loss-of-function variants is pLOF recessive = 1 for both genes.

Currently, the most accurate method for diagnosing Alport syndrome is comprehensive parallel genetic testing of the entire coding sequences of the *COL4A3, COL4A4*, and *COL4A5* genes using next-generation sequencing (NGS). The diagnostic utility is estimated at 80% if the disease is clinically suspected due to hematuria or progressive CKD, along with a positive family history of hematuria or ESRD [1].

Although a lot of *COL4A3, COL4A4,* and *COL4A5* variants have been revealed so far, novel changes and a large number of VUSs are still being identified and require a detailed evaluation to classify them as disease-causing alterations. This study aimed to establish the genetic background in a group of Polish patients with a clinical suspicion of Alport syndrome to enhance our understanding of the disease’s etiology (including molecular profile), epidemiology, and symptomatology in this population.

## 2. Materials and Methods

### 2.1. Patients

A group of 247 Polish individuals, referred between January 2015 and December 2023 to the Department of Medical Genetics of the Children’s Memorial Health Institute (CMHI), underwent genetic testing. In total, 138 unrelated probands, suspected with Alport syndrome based on clinical course, laboratory findings, and/or family history, along with their 109 family members, were included. The recruitment criteria for probands, derived from the Human Phenotype Ontology (HPO) database (https://hpo.jax.org/), encompassed symptoms selected from the primary five terms: hematuria (HP:0000790), proteinuria (HP:0000093), chronic kidney disease (HP:0012622), sensorineural hearing impairment (HP:0000407), abnormalities of the eye (HP:0000478), and additional hypertension (HP:0000822).

### 2.2. Genetic Testing and Data Analysis

Genetic testing for all 247 individuals was performed using genomic DNA samples automatically extracted from the peripheral blood leukocytes with a MagNA Pure LC 2.0 (Roche Diagnostics, Risch-Rotkreuz, Switzerland) or a MagCore Nucleic Acid Extractor HF16Plus (RBC Bioscience, New Taipei City, Taiwan) according to the manufacturer’s protocol.

#### 2.2.1. Next-Generation Sequencing and Variant Interpretation

Next-generation sequencing (NGS) was performed with the original CMHI NGS panel of >1000 clinically relevant genes (Roche Diagnostics, Risch-Rotkreuz, Switzerland, and Twist Bioscience, San Francisco, CA, USA) (Supplementary Table S1) or a TruSight One Sequencing Panel (Illumina, San Diego, CA, USA), both encompassing *COL4A3, COL4A4,* and *COL4A5* genes. Enriched libraries were paired-end-sequenced (2 × 100 bp) on the HiSeq 1500 or NovaSeq 6000 (Illumina, San Diego, CA, USA) according to the manufacturer’s protocol. The average read depth was 130, with >95% of the target regions covered at a depth of 20-fold.

Raw FASTQ reads were mapped to a slightly modified human genome assembly GRCh38/hg38, where duplicated regions were masked [12] using BWA [13]. Variant calling was performed using multiple algorithms (GATK HaplotypeCaller [14], MuTect2, FreeBayes [15], and DeepVariant [16]) to improve sensitivity for SNV, MNP, and small InDel detection, as well as to identify mosaic changes. Structural variants were identified using Lumpy [17], and CNV analysis was carried out using both CNVkit [18] and Decon [19]. The following quality control parameters were reported to evaluate the performance of the variant calling assessment: sensitivity 99.5%, precision >99%, and F-Score >99%. These reflect the experimentally validated results from our analysis pipeline. Alignments were visualized with the Integrative Genomics Viewer (IGV, https://igv.org/) [20].

Variant consequence annotation was performed using VEP [21], and the changes were further annotated with multiple data repositories, including the following:

- Frequency databases, such as the Genome Aggregation Database (gnomAD v4.1.0, https://gnomad.broadinstitute.org), and an in-house database with allele frequencies specific to the Polish population, comprising data from more than 12,000 individuals suspected of having a rare genetic disease (POLdb). According to the guidelines, the minor allele frequency (MAF) cutoff of 1% was adjusted, excluding known likely pathogenic and pathogenic variants, to account for the increased carriage frequency of pathogenic variants in Alport syndrome [1].
- Predicted impact on protein structure and function, assessed with in silico tools, including machine learning meta-scores (BayesDel, REVEL) and individual predictors (AlphaMissense, CADD, EIGEN, FATHMM-MKL, MutationTaster, PolyPhen-2, SIFT). Initially, REVEL was used for missense variants, with BayesDel applied if required, and their results were further supported by additional predictive tools. Variants occurring at splice sites were initially analyzed using SpliceAI, with additional evaluation supported by ADA, MaxEntScan, Pangolin and RF to assess their potential impact on normal splicing. Results from in silico tools were retrieved through the GeneBe (https://genebe.net/ (accessed on 1 December 2024)) and VarSome v12.9.0 (https://varsome.com/ (accessed on 1 December 2024)) platforms. Additionally, the Alamut Visual^™^ Plus software v1.3 (https://www.sophiagenetics.com/platform/alamut-visual-plus/ (accessed on 1 December 2024)) was used. Conservation was assessed using phyloP100 scores, which provide a quantitative measure of nucleotide or regional conservation relative to expectations under a neutral evolutionary model. Thresholds for predictive tool values were derived from the ClinGen Sequence Variant Interpretation Working Group’s recommendations [22,23].
- Occurrence in other reference databases, such as ClinVar (https://www.ncbi.nlm.nih.gov/clinvar (accessed on 1 December 2024)), the Leiden Open Variation Database (LOVD, https://www.lovd.nl/ (accessed on 1 December 2024)), and the Human Gene Mutation Database (HGMD, www.hgmd.cf.ac.uk (accessed on 1 December 2024)). The novelty of the detected variants was assessed using the aforementioned databases.

Finally, variants were interpreted based on the guidelines of the American College of Medical Genetics and Genomics and the Association for Molecular Pathology (ACMG/AMP) [24,25], and the Association for Clinical Genomic Science (ACGS) [26], in the context of Alport syndrome and the *COL4A3, COL4A4,* and *COL4A5* genes, as discussed in the Chandos House meeting of the Alport Variant Collaborative (AVC) [1,6]. ACMG/AMP criteria (pathogenic: very strong (PVS), strong (PS), moderate (PM), supporting (PP); and benign: stand-alone (BA), strong (BS), supporting (BP)) were assigned using an original online platform, GeneBe [27], followed by manual adjustments to certain criteria that could not be processed automatically, ensuring a more accurate assessment of pathogenicity. According to the criteria, benign and likely benign variants were filtered out, thereby retaining only pathogenic, likely pathogenic changes and variants of uncertain significance (VUS). The VUSs underwent detailed evaluation, with a particular focus on carrier analysis in the probands’ parents and on collecting information about symptoms in their family members. Finally, only VUSs with a pathogenicity score of 3 or higher were considered, as they were more likely to be classified as likely pathogenic based on genotype–phenotype correlation and assessment of carrier status.

The criteria for classifying variants, adjusted based on the consensus statement for Alport syndrome [1,6], are outlined below:

- PVS1 (Null Variant Evidence): Variants with a functional effect, such as nonsense, frameshift, or canonical ±1 or 2 splice site changes, which are mostly predicted to result in nonsense-mediated decay (NMD), are considered very strong pathogenic, with loss of function (LOF) being a known mechanism causing Alport syndrome. Non-canonical splice sites, as well as synonymous variants within the coding region predicted to result in splicing changes, have recently been indicated as disease-causing [1]. Additionally, glycine codon substitutions in *COL4A5* gene can disrupt splicing, especially when the change occurs at the last nucleotide of an exon [5].
- PS4 (Prevalence in Case–Control Study): This criterion is applied when a variant is found with higher frequency in individuals with Alport syndrome compared to controls. However, pathogenic variants in *COL4A3*, *COL4A4*, and *COL4A5* genes may appear in reference databases due to gender-specific (*COL4A5* variants) or age-dependent penetrance, as well as variable expression, with some individuals carrying pathogenic *COL4A3* and *COL4A4* monoallelic variants remaining asymptomatic or presenting only isolated hematuria.
- PM1 (Hotspot/Functional Domain): Variants affecting critical residues involved in the structure of collagen IV disrupt its normal production, which is essential for the glomerular basement membrane in the kidney, making these variants pathogenic and strongly indicative of disease. Most glycine residues in the intermediate collagenous domain (Gly-Xaa-Yaa repeats) are recognized as critical, equivalent to the well-established functional domain, as well as cysteine residues in the carboxy-terminal non-collagenous domain.
- PM2 (Population Frequency): Variants that are absent or rare in population databases are classified as pathogenic. However, the carrier frequency of pathogenic variants ranges from approximately 1 in 5000 for the *COL4A5* gene to 1 in 100 for the *COL4A3* and *COL4A4* genes. Additionally, hypomorphic variants or changes associated with a milder phenotype are increasingly recognized. Incomplete penetrance and variable expression of Alport syndrome often result in delayed recognition until substantial kidney dysfunction occurs. This indicates that pathogenic variants can also be present in reference databases of ostensibly healthy individuals.
- PM3 (Autosomal Recessive Inheritance): For the *COL4A3* and *COL4A4* genes, homozygous or compound heterozygous variants are classified as pathogenic. Parental testing is required to confirm whether the variants are in trans or to verify homozygosity.
- PM5 (Novel Missense Variant at a Known Pathogenic Site): This criterion is applied to novel missense variants occurring at codons previously associated with pathogenic changes. In the context of Alport syndrome, glycine substitutions in the Gly-Xaa-Yaa sequence are given particular weight due to their critical role in collagen IV stability. If a novel substitution occurs at such sites, the variant is classified as likely pathogenic or pathogenic based on its impact and existing evidence.
- PP1 (Segregation with Disease): Evidence for pathogenicity is supported by the segregation of variants in families with a history of Alport syndrome, particularly when the variant is consistently present in affected individuals but absent in unaffected family members.
- PP2 (Missense Variant in Conserved Region): For missense variants, conservation analysis is applied to determine the functional impact on the collagen structure. The collagen IV *α*3, *α*4, and *α*5 chains are highly conserved, particularly those of the glycine residues in the intermediate collagenous domains and many residues in the carboxy-terminal non-collagenous domains. In this regard, missense variants are a common mechanism for disease associated with the *COL4A3*, *COL4A4*, and *COL4A5 genes,* with a low rate of benign missense variation.
- PP3 and BP4 criteria (Supporting Pathogenic and Benign Evidence, respectively) are assigned based on in silico predictions from computational tools, as outlined above. Variants predicted to be benign by multiple algorithms are considered with reduced significance, recognizing that pathogenic variants in *COL4A3*, *COL4A4*, and *COL4A5* genes typically correlate with phenotype. These genes encode essential components of collagen IV, and pathogenic variants frequently result in significant structural and functional disruption, manifesting in clinically evident disease.
- PP4 (Phenotypic Specificity for Alport Syndrome): This criterion is applied when the observed phenotype closely matches the disease, particularly in individuals presenting with a combination of features such as hematuria, proteinuria, chronic kidney disease, sensorineural hearing impairment, and characteristic ocular findings, and/or a family history. The scoring scale applied for this criterion is as follows: 1 point for 2 symptoms, 2 points for 3 symptoms, and 4 points for 4 or 5 (>80%) symptoms presented in the proband. It is estimated that up to 80% of individuals with inherited hematuria carry a pathogenic variant in the *COL4A3*, *COL4A4*, and *COL4A5* genes.

The remaining pathogenicity criteria (PS1, PS2, PS3, PM4, PM6, BP7, BS1, BS3, BS4) were applied in accordance with the standard ACMG/AMP framework, without any specific adjustments for Alport syndrome. Notably, the PP5 criterion was excluded based on recent recommendations, as it relied on variant classification in public databases, which can be inaccurate or based on incomplete evidence. Additionally, certain benign-supporting criteria were omitted due to their limited relevance to Alport syndrome. These exclusions account for the genetic heterogeneity, including the prevalence of pathogenic and hypomorphic variants (BA1), incomplete penetrance and variable expression (BS2), differing inheritance patterns (BP2), various disease-causing variant types (BP1, BP3), and the potential for pathogenic collagen IV gene variants to exacerbate symptoms in other kidney disorders (BP5) [1,5,6,24,25,26].

#### 2.2.2. Sanger Sequencing

Segregation analysis was performed in available family members to validate variants considered as (potentially) disease-causing, identified in probands through NGS. This analysis was conducted using bi-directional Sanger sequencing with the BigDye Terminator v3.1 Kit (Applied Biosystems, Waltham, MA, USA) on the ABI 3130 Genetic Analyzer (Applied Biosystems, Waltham, MA, USA), according to the manufacturer’s protocol.

The nomenclature of molecular variants followed the Human Genome Variation Society (HGVS) guidelines (https://hgvs-nomenclature.org/) and referred to the MANE Select transcript [28], which aligns with the GRCh38/hg38 reference genome assembly. Accordingly, *COL4A3*, *COL4A4*, and *COL4A5* variants were described using the reference sequence for the collagen IV *α*3 chain (NM_000091.5, NP_000082.2), *α*4 chain (NM_000092.5, NP_000083.3), and *α*5 chain isoform 2 comprising 53 exons (NM_033380.3, NP_203699.1). Data regarding the complete primary structure of the human collagen IV *α*3, *α*4, and *α*5 chains were obtained from the original reports [1,29,30,31].

#### 2.2.3. Statistical Analysis

Continuous variables were expressed as the median (interquartile range, IQR). Allele frequency distributions were determined between the Alport syndrome and control (gnomAD European non-Finnish) population groups using the chi-square test. The odds ratio (OR) with 95% confidence interval (95% CI) was used to calculate the risk associated with specific alleles. A *p*-value of < 0.05 was considered statistically significant.

## 3. Results

### 3.1. Clinical Characteristics of Patients

The detailed clinical characteristics of our study group are provided in Supplementary Table S2, comprising 247 individuals from 138 families.

Clinical suspicion of Alport syndrome was established in 138 unrelated probands at a median age of 6 (IQR, 3–12). The median age at genetic testing was 13 years (IQR, 7–16), with a median interval of 3 years (IQR, 0–8) between clinical diagnosis and genetic testing. The most common clinical manifestation observed before probands’ referral was hematuria, present in 99% (137/138), followed by proteinuria in 51% (68/133). Notably, 68 probands exhibited both hematuria and proteinuria. A family history of hereditary kidney disease was present in most probands, accounting for 81% (112/138). Among 109 family members, the median age at genetic testing was 39.5 years (IQR, 17–45). This group included 73 parents and 36 other relatives.

Among the 247 individuals, i.e., probands and family members, 140 (57%) were females (F) and 107 (43%) were males (M). Hematuria was the most common clinical manifestation, observed in 81% (196/241; 137 probands and 59 family members). Proteinuria was detected in 40% (89/220; 68 probands and 21 family members), and 72% (108/151; 95 probands and 13 family members) had developed chronic kidney disease. A total of 24 probands and 2 family members had undergone kidney biopsy by the time of genetic testing. Sensorineural hearing impairment and ocular abnormalities were present in 16% (31/192; 21 probands and 10 family members) and 24% (47/194; 31 probands and 16 family members) of the individuals, respectively. Ocular symptoms included myopia, astigmatism, and retinal thinning. Hypertension was observed in 10% (18/188; 9 probands and 9 family members).

### 3.2. Molecular Findings and Genotyping

The genetic background was established in 109 out of 138 unrelated probands, corresponding to an overall diagnostic yield of 79%.

**Figure 1:**
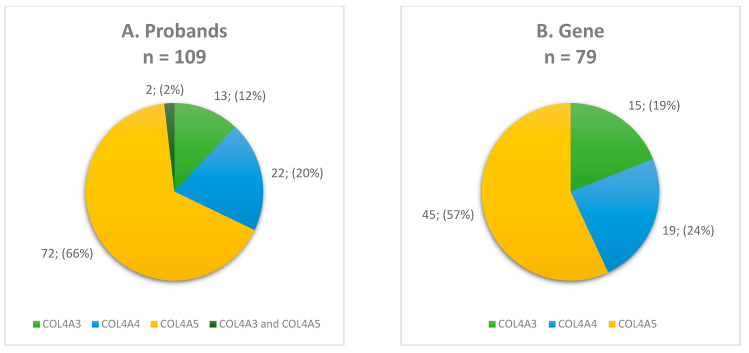

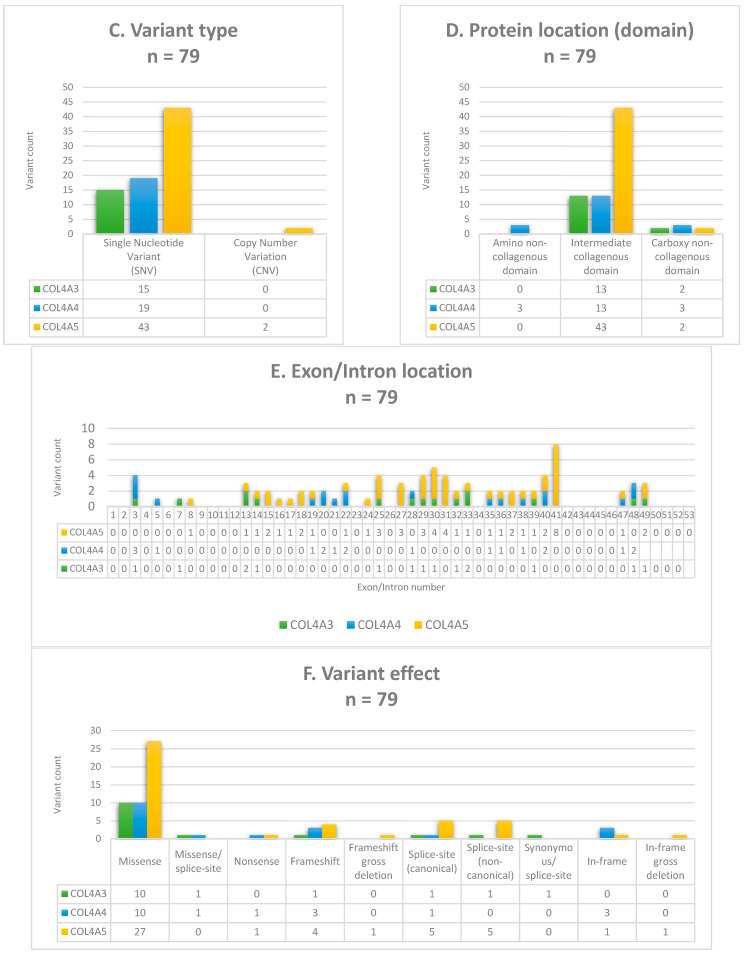

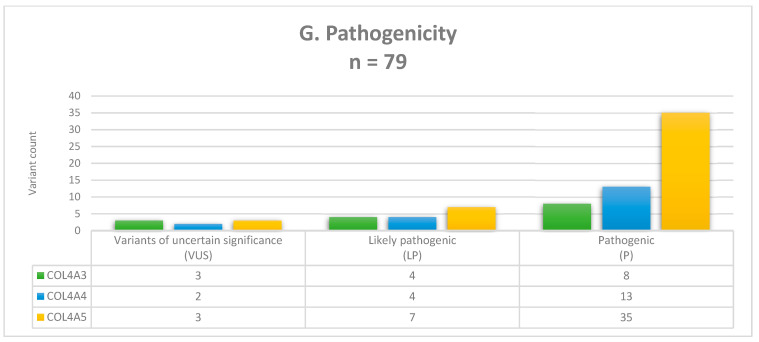
The proportions of (**A**) probands and (**B**–**G**) variants for each characteristic. The total number of (**A**) probands is 109, while the total number of (**B**–**G**) variants is 79.

Among these 109 probands (F = 62, M = 47), 72 (F = 40, M = 32) carried variants exclusively in the *COL4A5* gene, highlighting the predominance of X-linked inheritance in our study group. Of the remaining probands, 13 (F = 8, M = 5) were carriers of *COL4A3* changes, while 22 (F = 12, M = 10) carried variants in the *COL4A4* gene. Additionally, two female probands carried one variant each in *COL4A3* and *COL4A5* genes (digenic inheritance) (Figure 1A).

In total, 63% of 109 probands were monoallelic heterozygotes for variants in the *COL4A3* (*n* = 10), *COL4A4* (*n* = 19), and *COL4A5* (*n* = 40) genes, while 29% (*n* = 32) were hemizygotes for the *COL4A5* gene. Two different heterozygous variants in the *COL4A3* or *COL4A4* gene were identified in 6% of probands (*n* = 6) (F017, F030, F036, F043, F068, and F131). Carrier testing of both parents was possible in only three of these families (F017, F036, and F043), confirming the biallelic occurrence of variants and suggesting the diagnosis of the recessive form of Alport syndrome. Two probands (2%) (F060 and F065) each carried one heterozygous variant—c.4523A>G or c.4421T>C in the *COL4A3* gene and c.1871G>A in the *COL4A5* gene—following a digenic inheritance pattern. No homozygous predicted pathogenic, likely pathogenic changes were revealed.

Overall, 79 different *COL4A3, COL4A4*, and *COL4A5* variants were revealed in our study group. The detailed characteristics of identified molecular variants are provided in Supplementary Table S3. The *COL4A5* gene accounted for more than half of the identified variants (57%), while 19% and 24% were located in the *COL4A3* and *COL4A4* genes, respectively (Figure 1B). Regarding variant zygosity, 72% of variants were heterozygous in the *COL4A3, COL4A4*, and *COL4A5* genes and 24% were hemizygous in the *COL4A5* gene. An additional three (4%) changes were identified in both the heterozygous and hemizygous states in the *COL4A5* gene.

About 97% of the changes were single-nucleotide variants (SNVs). Only two copy number variations (CNVs) were identified (Figure 1C). Both deletions were hemizygous and located within the intermediate collagenous domain of the *COL4A5* gene. The first was a multi-exon deletion c.(780+1_781−1)_(1165+1_1166−1)del (containing exons 14–19) that disrupted the reading frame and was predicted to result in NMD, while the second, a single-exon 22 deletion c.(1423+1_1424−1)_(1516+1_1517−1)del, was an in-frame variant that preserved the reading frame, removed less than 10% of the protein, and did not result in NMD. The exon 22 deletion was novel, and carrier testing indicated that this variant was maternally inherited, which correlated with the family history (F073), thus enhancing its pathogenicity. Both CNVs were absent in gnomAD and classified as pathogenic and likely pathogenic, respectively.

Among the SNVs, missense substitutions were the most common (60%) in our study group. In 85% of these variants, glycine was the substituted amino acid, while the remaining 15% involved substitutions of other residues, such as Arg, Asn, Cys, Leu, and Pro. A total of 80% of glycine substitutions involved highly destabilizing residues (Glu, Arg, Asp, Val) and mildly destabilizing residues, such as Ala, Ser, Cys, were also observed.

The remaining SNVs included nonsense (*n* = 2), frameshift (*n* = 8), canonical (*n* = 7) and non-canonical (*n* = 6) splice site as well as in-frame (*n* = 4) changes. Additionally, the three variants outlined below, initially involving the alteration of a non-conserved nucleotide and classified as missense or synonymous, were assessed in silico for their functional consequences and identified as potential splice site variants (Figure 1F). The missense *COL4A3*:c.3416C>T variant, located three nucleotides upstream from the end of the exon 39 in the intermediate collagenous domain, was predicted to result in the loss of donor splice sites by SpliceAI (score = 0.71), Pangolin (score = 0.47), and ADA (score = 1). Another missense variant, *COL4A4*:c.1100G>T, is located at the first nucleotide of the exon 19, near a canonical splice site and adjacent to a non-collagenous interruption in the intermediate collagenous domain. SpliceAI (score = 0.21) and Pangolin (score = 0.13) weakly predicted acceptor loss for this variant; however, ADA (score = 0.99) and RF (score = 0.88) indicated significant alterations in normal splicing. Both variants were absent in gnomAD, but were observed in POLdb, with no available phenotype information. The synonymous *COL4A3*:c.441G>A variant is located at the last nucleotide of the exon 7 in the intermediate collagenous domain and was predicted by SpliceAI (score = 0.70), Pangolin (score = 0.67), ADA (score = 1), and RF (score = 0.93) to affect mRNA splicing, leading to a significantly altered protein sequence by abolishing the canonical donor splice site. The variant allele was found at a frequency of 0.00004034 in gnomAD, with no homozygous occurrence. All three variants were previously reported in the ClinVar or LOVD databases, and their significance was classified as VUS or likely pathogenic. In our study group, the identified variants correlate with the symptoms observed in probands and their family members (F047, F083, and F102). This correlation was further supported by carrier analysis and the presence of these variants in symptomatic parents, providing additional evidence of their pathogenicity.

When classifying variants according to exon locations, 80% were identified between exon 21 and the carboxy-terminal non-collagenous domain, while the remaining 20% were located within amino-terminal non-collagenous domain and exon 20, regardless of the gene involved (Figure 1E). Although variants were distributed across all domains of collagen VI genes, the amino-terminal non-collagenous domain was the least affected, with only three *COL4A4* variants: c.81_86del, c.93_94del, and c.112G>C. Seven variants were identified in the carboxy-terminal non-collagenous domains of *COL4A3*, *COL4A4*, and *COL4A5* genes. The intermediate collagenous domain was the most affected, with 87% of the identified variants located within it (Figure 1D). The intermediate collagenous domain contains non-collagenous interruptions within the Gly-Xaa-Yaa repeats, where three SNVs were identified, *COL4A3*:c.744_765+66del, and *COL4A4*:c.2286_2292delinsGGGACCAG, c.3242_3243del. Additionally, two CNVs in the *COL4A5* gene, outlined above, encompassing the non-collagenous interruptions, were found. In our study group, four variants adjacent to non-collagenous interruptions were also identified: *COL4A4*:c.1100G>T, and *COL4A5*:c.1780G>A, c.1871G>A, c.4114G>T.

Most of the identified variants were unique to the studied group; however, 11 recurrent variants were observed in unrelated probands. Six variants appeared twice: *COL4A3*:c.4421T>C, *COL4A4*:c.3743G>A, and *COL4A5*:c.458G>A, c.2414G>T, c.2723G>A, c.3399delA. Two variants appeared three times: *COL4A3*:c.2083G>A, *COL4A4*:c.1396G>A. The variants *COL4A4*:c.3734G>T and *COL4A5*:c.2350G>C were identified four and five times, respectively. Moreover, the *COL4A5*:c.1871G>A variant was revealed in 23 probands, with an overall allele frequency of 13.4%, corresponding to 23 out of a total of 171 investigated alleles. It also accounted for 31% (23/74) of all *COL4A5* affected alleles.

The two identified changes are known hypomorphic variants: *COL4A3*:c.4421T>C and *COL4A5*:c.1871G>A. The *COL4A3*:c.4421T>C appeared twice. In one proband (F068), it co-occurred with *COL4A3*:c.744_765+66del, but the allelic origin could not be determined due to the unavailability of parental testing. However, the family history was positive for symptoms associated with the disease. In the second proband (F065), *COL4A3*:c.4421T>C occurred alongside *COL4A5*:c.1871G>A, and digenic inheritance was considered. The family history suggested a maternal origin for both variants, which was subsequently confirmed through carrier analysis. The proband’s sister carried only the *COL4A3*:c.4421T>C variant and also developed hematuria. Regarding the *COL4A5*:c.1871G>A variant, it was identified in 23 probands. In addition to the above-described proband (F065), digenic inheritance was also considered in another proband (F060), where the *COL4A5*:c.1871G>A variant occurred together with the *COL4A3*:4523A>G variant. The family history was significant for CKD, but only the asymptomatic brother was available for genetic testing, and no changes were detected in the results. The remaining 21 probands (F = 11, M = 10) carried only the *COL4A5*:c.1871G>A variant and developed hematuria, with or without proteinuria, and extrarenal manifestations (Supplementary Table S2).

Among the identified variants, 23 were novel, while 56 were previously reported in databases such as ClinVar, HGMD, or LOVD. The pathogenicity status of all 79 variants was evaluated as described in the methods of this study. A total of 71% (56/79) of variants were classified as pathogenic, and 19% (15/79) were considered likely pathogenic. Only eight (10%) were classified as VUSs: *COL4A3*:c.166C>T, c.4421T>C, c.4523A>G, *COL4A4*:c.81_86del, c.112G>C, and *COL4A5*:c.2435C>T, c.3107-4A>G and c.3715C>G (Figure 1G). The details are outlined below.

The missense *COL4A3*:c.166C>T p.(Pro56Ser) variant involves the alteration of a non-conserved nucleotide (PhyloP100 = 1.00) within the intermediate collagenous domain. The variant allele was found at a frequency of 0.000001864 in gnomAD, with no homozygous occurrences, and was reported only in dbSNP (rs1204689662) without accompanying clinical assessments. REVEL indicated indeterminate pathogenicity (score = 0.342), while only the PolyPhen-2 tool supported pathogenic classification. This variant was identified in a proband (F134) with isolated hematuria and no family history; carrier analysis was not performed. This variant was classified as a VUS, with 4 points according to ACMG/AMP criteria, indicating that it has a 67.5% probability of a negative effect on the protein (posterior probability = 67.5%).

The *COL4A3*:c.4421T>C p.(Leu1474Pro) variant involves the alteration of a conserved nucleotide (PhyloP100 = 7.674) within the carboxy-terminal non-collagenous domain. This hypomorphic substitution has been previously reported with a conflicting classification of pathogenicity in the ClinVar, LOVD, and HGMD databases. REVEL classified this variant as moderately pathogenic (score = 0.788). It was observed twice in our study group (F065 and F068), as above-described, and it was classified as a VUS, with 5 points according to ACMG/AMP criteria (posterior probability = 81.2%).

The *COL4A3*:c.4523A>G p.(Asn1508Ser) variant is a missense substitution located within the carboxy-terminal non-collagenous domain, involving the alteration of a conserved nucleotide (PhyloP100 = 7.16). This variant has been previously reported in the ClinVar and LOVD databases, where it was classified as a VUS. Its allele frequency in gnomAD was 0.0003724, with no homozygous occurrences reported, including 0.0004839 in the European (non-Finnish) population. In POLdb, the MAF was 0.0012, observed in individuals for whom mild symptomatic Alport syndrome could not be excluded. The variant co-occurred with *COL4A5*:c.1871G>A, suggesting a potential digenic inheritance pattern in a proband (F060) with chronic kidney disease and a family history of chronic kidney disease. Parental samples were unavailable for carrier testing, and the variant was absent in the asymptomatic brother. This variant was classified as a VUS, with 4 points, according to ACMG/AMP criteria.

The *COL4A4*:c.81_86del p.(Ile29_Leu30del) variant is predicted to remove two amino acids from exon 3 of the encoded protein while preserving the integrity of the reading frame. It is located within the amino-terminal non-collagenous domain. The allele frequency of this variant in gnomAD was 0.00005466, with no homozygous occurrences reported, including 0.00006461 in the European (non-Finnish) population. This in-frame deletion has been reported with conflicting pathogenicity classifications in the ClinVar, LOVD, and HGMD databases and has been associated with both autosomal recessive and dominant forms of disease. In our study group, it was identified in a heterozygous state in an affected proband (F078) with a family history of inherited kidney disease observed in the proband’s sister, maternal aunt, and maternal grandmother. Only unaffected parents were tested, and the variant was detected in the asymptomatic mother. The clinical variability in this family may be attributed to the incomplete penetrance associated with Alport syndrome. Performing carrier testing, particularly in affected family members, could provide valuable data to clarify the pathogenicity of this variant. Careful interpretation of variants demonstrating incomplete penetrance or variable expression is essential in such cases. Currently, this variant has been classified as a VUS, scoring 5 points, according to the ACMG/AMP criteria.

The *COL4A4*:c.112G>C p.(Gly38Arg) variant is a novel missense change involving the alteration of a non-conserved nucleotide (PhyloP100 = 3.332) within the amino-terminal non-collagenous domain. The glycine residue was replaced by arginine, considered a highly destabilizing amino acid. The variant was absent from gnomAD. Although located near the splice region, in silico tools predicted no significant impact on normal splicing. REVEL and BayesDel did not provide supporting evidence, while CADD, FATHMM-MKL, MutationTaster, PolyPhen-2, and SIFT predicted pathogenicity. Variant co-segregated with the disease in the family and has maternal origin (F022). Identification of this variant in another affected individual further enhances its pathogenicity. According to the ACMG/AMP criteria, this variant was classified as a VUS, scoring 4 points.

The *COL4A5*:c.2435C>T p.(Pro812Leu) variant is a novel missense substitution located within the intermediate collagenous domain, involving the alteration of a non-conserved nucleotide (PhyloP100 = 2.53). The variant was absent from gnomAD. REVEL (score = 0.441) and BayesDel did not provide pathogenic supporting evidence, while only FATHMM-MKL and MutationTaster predicted pathogenicity. This variant was identified in an affected proband (F136) with a family history of hematuria. Parental samples were unavailable for carrier testing. According to the ACMG/AMP criteria, this variant was classified as a VUS, scoring 4 points.

The *COL4A5*:c.3107-4A>G p.? variant is located in a non-canonical splice site. It involves the alteration of a weakly conserved nucleotide (PhyloP100 = 3.353) within the intermediate collagenous domain. The variant was absent from gnomAD. SpliceAI (score = 1.0) and Pangolin (score = 0.85) predicted that this variant is associated with the activation of a cryptic splice site (acceptor gain) in intron 35, but is expected to preserve the integrity of the reading frame. Additionally, ADA (score = 0.99) and RF (score = 0.93) indicated significant alterations in normal splicing. This variant has been classified as likely pathogenic in ClinVar, although the related clinical condition has not been confirmed. In our study group, it was identified in an affected male proband (F059) with a family history of Alport syndrome in the mother and maternal grandfather. Family members were unavailable for carrier testing. This variant, currently classified as a VUS, with 5 points according to ACMG/AMP criteria, is likely to be classified as likely pathogenic after future assessment of carrier status and genotype–phenotype correlation.

The *COL4A5*:c.3715C>G p.(Pro1239Ala) variant is a novel missense change involving the alteration of a conserved nucleotide (PhyloP100 = 3.661) within the intermediate collagenous domain. The variant was absent from gnomAD. REVEL (score = 0.419) and BayesDel did not provide pathogenic supporting evidence, while only FATHMM-MKL, MutationTaster, and PolyPhen-2 supported pathogenic classification. Additionally, it occurred alongside the pathogenic *COL4A5*:c.3721G>A variant; however, current recommendations omit the use of the benign-supporting BP2 criterion for Alport syndrome. Their relatively close location within the same exon allowed for the visualization of the NGS results using the IGV software v2.16.2, which indicated that both variants were likely present in cis configuration. Carrier testing within this family was conducted only on the asymptomatic mother, and no changes were detected in the results. It was not possible to determine whether the variants were inherited or in cis, as testing could not be performed on the father, and his clinical status was unknown (F058). This variant was classified as a VUS, with 4 points, according to ACMG/AMP criteria.

### 3.3. Carrier Testing

Carrier testing was performed in 53% (58/109) of the families. In some families, only one parent was available for testing, and occasionally, other family members were tested instead. Variants were primarily inherited from affected parents or observed in affected family members. In three families, variants *COL4A5*:c.781-2A>G (F048), c.3604+1G>A (F008), and c.4822−16_4822−12del (F006) were assumed to be de novo, and there was no family history of Alport syndrome.

## 4. Discussion

In our study, we characterized the clinical and genetic features of 247 Polish individuals, i.e., probands and family members, from 138 families with clinically suspected Alport syndrome. Hematuria was the most prevalent clinical feature, observed in 81% of individuals in our study group. In addition to renal symptoms, some probands and their family members exhibited sensorineural hearing impairment, ocular abnormalities, and hypertension, consistent with classical manifestations of the disease. A family history of hereditary kidney disease was present in 81% of probands, emphasizing the genetic basis of Alport syndrome. The median ages at clinical diagnosis and genetic confirmation for probands were 6 and 13 years, respectively, with a median gap of 3 years between the two. This delay in genetic testing aligns with the incomplete penetrance and variable expression of Alport syndrome, which is often not fully recognized until substantial kidney dysfunction occurs. The median age of family members at the time of genetic testing was 39.5 years, highlighting delays in the molecular confirmation of their disease due to previously limited diagnostic options. Traditional Sanger sequencing of multi-exon genes such as *COL4A3*, *COL4A4*, and *COL4A5* was both time-consuming and costly. The introduction of next-generation sequencing has significantly reduced diagnostic process time and costs. Moreover, targeted Sanger sequencing for specific variants identified in probands now enables efficient confirmation or exclusion of carrier status among family members.

**Figure 2:**
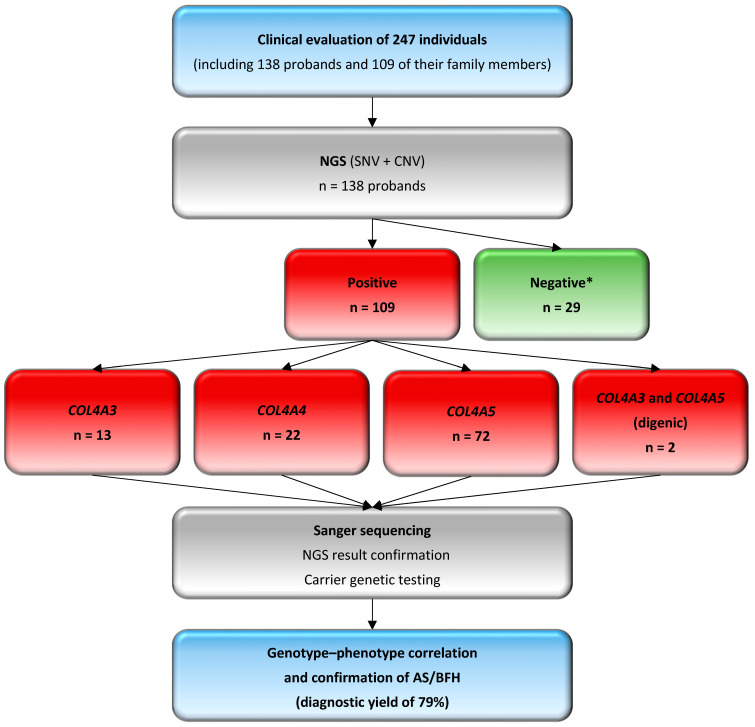
Flowchart of 138 probands with a clinical suspicion of Alport syndrome, along with their 109 family members. NGS, next-generation sequencing; CNV, copy number variation; SNV, single-nucleotide variant; AS, Alport syndrome; BFH, benign familial hematuria. * No pathogenic or likely pathogenic variants, nor variants of uncertain significance, were found in the remaining genes of the NGS panel comprising over 1000 clinically relevant genes (Supplementary Table S1) or in the TruSight One Sequencing Panel. Probands with negative results require differential diagnosis for other glomerulopathies.

This study achieved a diagnostic yield of 79%, revealing pathogenic, likely pathogenic changes, and VUS in 109 of 138 probands suspected with Alport syndrome. Our result correlates with the diagnostic utility reported in the literature, estimated at 80% [1]. A total of 79 variants were identified across all three collagen IV genes: *COL4A3*, *COL4A4*, and *COL4A5*. The majority (57%) were located in the *COL4A5* gene, reflecting the predominance of X-linked inheritance in Alport syndrome, while variants in the *COL4A3* (19%) and *COL4A4* (24%) genes accounted for autosomal forms of the disease (Figure 2).

Among the probands with a clinical suspicion of Alport syndrome and detected changes, we revealed monoallelic variants in the *COL4A3* or *COL4A4* gene in 27% (29/109). However, not all of these individuals could be classified as autosomal dominant Alport syndrome due to limited or conflicting clinical data. Furthermore, we preferred to avoid this term in accordance with recent recommendations and the lack of a clear consensus regarding the terminology for monoallelic carriers of variants in these genes [1]. These individuals may not only present with the dominant form of Alport syndrome, but also with a benign form of familial hematuria, or may simply be carriers of the recessive form of Alport syndrome. Heterozygotes with a pathogenic *COL4A3* or *COL4A4* variant have hematuria, and sometimes proteinuria, kidney impairment, and hypertension, but they rarely have sensorineural hearing impairment or ocular abnormalities [1]. In our probands, all 29 monoallelic for *COL4A3* or *COL4A4* presented with hematuria, and 6 of them had isolated hematuria. None had isolated proteinuria, while four exhibited both hematuria and proteinuria without CKD. However, CKD was observed in 19 probands with both hematuria and proteinuria. Additionally, one proband had hypertension, two had sensorineural hearing impairment, and four had ocular abnormalities. Regarding their family members, 90% of families had a history of hereditary kidney disease, often accompanied by extrarenal manifestations, which presented in various combinations. In eight probands, the family history was limited to hematuria, which may suggest a benign form of familial hematuria. Nevertheless, it is recommended to perform carrier testing within families of probands with heterozygous pathogenic *COL4A3* or *COL4A4* variants due to their risk of impaired kidney function. Experts also recommend that *COL4A3* or *COL4A4* heterozygotes should not act as kidney donors [1].

The identification of biallelic variants confirming autosomal recessive inheritance in three families undergoing carrier testing, as well as evidence of potential digenic inheritance in two probands, further emphasizes the genetic heterogeneity and complexity of Alport syndrome. Increasingly, digenic inheritance is being reported. The most common form of digenic Alport syndrome involves pathogenic variants in both *COL4A3* and *COL4A4* genes, potentially resulting in a more severe clinical phenotype compared to individuals with a single variant. However, individuals with digenic variants in *COL4A5* and *COL4A3* or *COL4A5* and *COL4A4* are also at risk of kidney impairment [1]. In our study group, both probands with variants in *COL4A5* and *COL4A3* had a family history of chronic kidney disease. One female proband and her mother (F065) presented with hematuria only, while her brother exhibited both hematuria and proteinuria, which may reflect the more severe phenotype typically observed in males with *COL4A5* variants [1]. Interestingly, another female proband (F060) had only chronic kidney disease at the age of 11 and was referred for genetic testing due to their family history.

Among the 79 identified variants, the majority were single-nucleotide variants (97%), with missense changes being the most common (60%). A significant proportion (85%) involved glycine substitutions, which are known to disrupt the collagen IV structure. Additionally, in silico splicing predictions further refined variant classifications, revealing potential splice defects in variants initially annotated as synonymous or missense. Only two copy number variants were detected, both in the *COL4A5* gene. One multi-exon deletion likely triggered NMD, while the single-exon deletion preserved the reading frame, demonstrating the varying effects of structural changes.

A large number of variants were located in the intermediate collagenous domain, which is particularly relevant given its critical role in the structural integrity of the glomerular basement membrane. The results outlined above, regarding the frequency of changes in individual genes, types of variants, glycine amino acid substitutions, and the distribution of variants within domains, support the findings of previous studies [1].

The presence of recurrent variants in unrelated individuals further underscores the pathogenic potential of these changes. The most frequent variant, *COL4A5*:c.1871G>A p.Gly624Asp (commonly known as p.G624D), was identified in 21% (23/109) of probands with changes detected in the *COL4A3*, *COL4A4*, and *COL4A5* genes. The allele frequency of this variant was 13.4% (23 out of a total of 171 alleles) and it accounted for 31% (23/74) of all affected *COL4A5* alleles. This variant was even more frequent in another study of 269 Polish children enrolled into a National Registry for children with persistent glomerular hematuria, where the *COL4A5*:c.1871G>A variant was the most prevalent, accounting for 39% (44/113) of genetically confirmed X-linked Alport syndrome in unrelated Polish families, thus making it the predominant variant in this national cohort [7]. Originating in the Middle Ages, *COL4A5*:c.1871G>A is predominant in Central and Eastern Europe and is known as a hypomorphic variant. In the general population (gnomAD), the allele frequency of the *COL4A5*:c.1871G>A variant was 0.00004629, with no homozygous occurrences reported, and it was the most frequent (AF = 0.00004833) in the European (non-Finnish) population, including 23 hemizygotes (population Grpmax Filtering AF with 95% confidence). Although the *COL4A5*:c.1871G>A variant occurs significantly more frequently in POLdb than in the gnomAD European (non-Finnish) population database (OR = 29.1341, 95% CI: 19.6432 to 43.2104, z-statistic = 16.767, *p* < 0.0001), its allele frequency (MAF = 0.00179) does not exceed 1%, which was the cutoff value for the variant prioritization. This variant is located adjacent to a non-collagenous interruption in the intermediate collagenous domain, which contributes to its mostly mild phenotype. Despite the benign clinical manifestation throughout childhood and early adulthood, this variant confers significant risk for both kidney impairment and sensorineural hearing impairment in males [7]. In our study group, *COL4A5*:c.1871G>A appeared in a substantial proportion (83%) of individuals with a family history of hereditary kidney disease. The majority of probands exhibited hematuria (96%), while 13 had proteinuria and 16 developed chronic kidney disease. Four of them also had sensorineural hearing impairment, as well as ocular abnormalities.

In our study group, the second known hypomorphic variant *COL4A3*:c.4421T>C was identified in two probands. This variant co-occurred with *COL4A3*:c.744_765+66del in one proband, but the allelic origin could not be determined due to the lack of parental testing. In another proband, it occurred together with the hypomorphic *COL4A5*:c.1871G>A variant and co-segregated with the family disease. The substitution *COL4A3*:c.4421T>C occurs in the carboxy-terminal non-collagenous domain. According to the literature, on its own, it may be associated with a normal urinary sediment, proteinuria, and focal segmental glomerulosclerosis or, in association with another *COL4A3* variant, with autosomal recessive Alport syndrome and kidney impairment [10]. The presence of hypomorphic variants highlights the need for careful interpretation of variants that show incomplete penetrance or variable expression.

The pathogenicity classification followed the ACMG/AMP guidelines adapted for Alport syndrome. The GeneBe platform was valuable in classifying variants by summarizing the results for ACMG/AMP criteria, such as the computational tools and occurrence in gnomAD and other reference databases. However, detailed clinical data and assessment of carrier status were crucial. Co-segregation analysis, especially of novel changes in families, proved to be a powerful tool for classifying these variants as potentially pathogenic. Variants were categorized as pathogenic (71%), likely pathogenic (19%), and VUS (10%). Among the 23 novel variants, 14 were identified as pathogenic, 5 as likely pathogenic, and 4 as VUS, respectively. The detailed scoring system for pathogenicity assessment is provided in Supplementary Table S3. A significant aspect of our findings was the identification of several changes classified as variants of uncertain significance. Three variants previously described in databases as VUS or with conflicting interpretations of pathogenicity (*COL4A3*:c.4421T>C, c.4523A>G, and *COL4A4*:c.81_86del) have been evaluated in our study, and we maintain this classification based on our assessment. These variants require further functional validation to confirm their involvement in the disease. Additionally, co-segregation analysis in families and the identification of the same variant in other affected individuals would further support its pathogenicity. Among the known variants previously reported as VUS or with conflicting interpretations of pathogenicity, according to our assessment, several have been reclassified as likely pathogenic (*COL4A3*:c.441G>A, c.3416C>T and *COL4A4*:c.3577+1G>A, c.4781_4807dup, c.5045G>A) or pathogenic (*COL4A3*:c.1594G>T, *COL4A4*:c.1396G>A, c.3307G>A, c.3743G>A, and *COL4A5*:c.2105G>T). This reclassification was influenced by a combination of factors, including detailed clinical and family history assessments, carrier testing, and the co-segregation of variants with the disease in multiple affected family members. Additionally, the identification of the same variant in more than one affected proband within our study group further supported the pathogenicity of these variants. Other contributing factors included the confirmation of the variant in trans with another pathogenic variant, indicating a recessive form of Alport syndrome; the identification in the literature of another pathogenic change at the same amino acid residue; and evidence showing that the prevalence of these variants in affected individuals is significantly higher compared to their prevalence in the general population (gnomAD European (non-Finnish) population database). Our comprehensive approach underscores the importance of integrating clinical and familial with molecular data to achieve accurate variant interpretation.

Our study emphasizes the importance of family history and carrier testing in diagnosing Alport syndrome. In many families, variants were inherited from affected parents, and carrier analysis provided valuable insights into the inheritance patterns. However, the identification of de novo variants in some families underscores the complexity of the inheritance and the potential for new variants to arise.

Our study had its own limitations, particularly in applying the ACMG/AMP criteria to variants where clinical data were unavailable, especially for family members. We also lacked carrier status analysis for 47% of the probands, and in some cases, only one parent or another family member was available for testing. In many cases, variants classified as VUS require further carrier testing to determine their pathogenicity, as well as testing in families where two variants in a single gene have been revealed to determine allelic origin, or in different genes to establish digenic inheritance. Although our approach may not have addressed all criteria, it allowed us to exclude benign and likely benign variants. However, the primary aim of this study was to characterize the molecular spectrum in a group of Polish patients, rather than to assess the detailed genotype–phenotype correlation in Alport syndrome. Due to the molecular and clinical heterogeneity of the disease, a larger study group and more comprehensive data collection are needed, and further studies are planned to address these aspects. There are also some future directions. Further investigation of the identified VUS variants will be essential to refine their pathogenicity and better understand their impact on the disease. Performing missing carrier testing, especially in high-risk families, may enable earlier interventions to delay CKD progression and is important for genetic counselling. Monitoring probands and identified carriers could provide valuable data on disease progression and the impact of various interventions.

Nevertheless, this study broadens the molecular spectrum of Alport syndrome. The identification of 23 novel variants indicates that there are still many potentially pathogenic variants in the *COL4A3*, *COL4A4*, and *COL4A5* genes that remain unknown. These variants, in particular, require detailed evaluation to classify them as disease-causing changes. Overall, we established that genetic confirmation of diagnosis is essential for diagnosing Alport syndrome. Traditionally, kidney biopsy was the primary diagnostic method; however, genetic testing provides a less invasive and more precise alternative, while also revealing the underlying pattern of inheritance and reducing the diagnostic process duration to no longer than 6 months.

## 5. Genetic Counselling

Our findings highlight the importance of the molecular investigation of Alport syndrome, including the most common *COL4A5*:c.1871G>A and *COL4A3*:c.4421T>C variants, in the Polish population, with critical implications for genetic counselling and clinical practice. The relatively high frequency of these variants (MAF = 0.00179 and MAF = 0.0024, respectively; Supplementary Table S3) underscores the need for targeted strategies in genetic counselling to address the inheritance patterns and clinical variability of Alport syndrome. According to our data and previously published findings, heterozygous carriers, unlike hemizygotes/compound heterozygotes, tend to exhibit a milder phenotype or may remain asymptomatic for years due to incomplete penetrance (e.g., F017, F036, F043, F093 in our study). Moreover, hypomorphic variants or changes associated with a milder Alport syndrome phenotype are increasingly being recognized. The incomplete penetrance and variable expression of Alport syndrome often result in delayed diagnosis, typically only after substantial kidney dysfunction has occurred, underscoring the importance of early genetic screening, monitoring, and regular follow-up of patients to detect and manage renal progression more effectively. This is also complicated by the presence of “mild” pathogenic variants in healthy individuals’ reference databases. Furthermore, the identification of two hypomorphic variants in the Polish population, with a combined frequency of up to 0.44%, and their potential occurrence in digenic configurations emphasizes the complexity of genetic risk assessment and the necessity of precise reproductive counselling, especially for milder phenotypes that may remain undetected for years. Although some cases exhibit non-penetrance, the associated risk remains higher than in other rare diseases. Clinicians must carefully assess risks for offspring and grandchildren of affected individuals, as the lifetime risk of developing renal symptoms in children of patients with severe pathogenic variants differs markedly from typical recessive or dominant Mendelian disorders. The integration of NGS into clinical practice has introduced both opportunities and challenges in genetic diagnostics, particularly in the interpretation of test results and incidental findings, as well as technical limitations in the event of inconclusive results. The advantages of molecular diagnostics are particularly evident in female carriers of Alport syndrome, where distinguishing between a heterozygous variant in the X-linked *COL4A5* gene and a heterozygous variant in *COL4A3* and *COL4A4* genes has significant implications for genetic counselling, especially in prenatal contexts, and long-term prognosis. For carriers, specific counselling is essential, including the recommendation against kidney donation due to the risk of progressive renal damage. This distinction informs the management of renal and extrarenal phenotypes and evaluates risks such as post-transplant anti-GBM nephritis, demonstrating that genetic testing can provide a diagnosis and guide prognostic and therapeutic decision-making in patients with Alport syndrome. Despite the advantages of molecular diagnostics, the interpretation of VUS remains challenging, as these variants may mimic disease-causing changes but lack sufficient evidence for definitive classification. Therefore, the periodic re-evaluation of such variants, alongside functional studies and co-segregation analyses, is essential for refining their pathogenicity and providing patients with up-to-date information. This approach ensures that individuals and their families receive accurate information and appropriate care strategies based on their genetic risk.

Centralized patient care, encompassing clinical evaluation, genetic testing, and genetic counselling within a single health institution, provided substantial advantages in both diagnostic accuracy and patient management. This integrated approach ensured standardized diagnostic protocols, reduced variability in test interpretation and clinical assessments, and facilitated the delivery of personalized genetic counselling. Probands and their families received clear guidance on their health condition, inheritance patterns, and the available options for preventing disease progression. Additionally, this centralized approach promoted multidisciplinary collaboration among clinicians, geneticists, molecular biologists, and bioinformaticians. This enhanced care quality and fostered a robust foundation for research and evidence-based practice. This model also strengthened the ability to correlate clinical phenotypes with genetic findings, contributing to a better understanding and management of Alport syndrome.

## 6. Conclusions

In conclusion, the use of an NGS panel has shown considerable promise in the field of Alport syndrome, increasing the diagnostic rate to 79% and reducing the time to diagnosis. The phenotype-driven gene panel, which is specific for genetic diseases in the pediatric population, is an affordable alternative to WGS and WES, offering comparable diagnostic efficacy and supporting its implementation as a first-line genetic test in rare diseases, including Alport syndrome. Our study provides important insights into the genetic landscape of Alport syndrome, confirming the central role of *COL4A5* variants in X-linked inheritance and the involvement of *COL4A3* and *COL4A4* genes in autosomal forms. Digenic inheritance in some probands further emphasizes the genetic heterogeneity and complexity of Alport syndrome. The high diagnostic yield of genetic testing underscores its importance in the clinical management of the disease, while the identification of novel and recurrent variants enhances our understanding of the genetic basis of Alport syndrome. The high frequency of the *COL4A5*:c.1871G>A variant in Alport syndrome probands (13.4%) as well as in Polish population of rare diseases (0.2%), along with its overall frequency, underscores the importance of considering *COL4A3*, *COL4A4*, and *COL4A5* genes for inclusion in the list of secondary findings. This approach would facilitate the identification of individuals potentially affected with Alport syndrome, enabling early diagnosis and the introduction of renoprotective treatment to prevent progression to chronic kidney disease and end-stage renal disease. Revealing variants of uncertain significance highlights the need for continued research to refine genetic testing criteria and improve diagnostic accuracy. Based on the obtained genotype–phenotype correlation, we assessed that NGS allows us to avoid invasive renal biopsy in Alport syndrome diagnosis. It provides the disease confirmation or exclusion, identification of atypical Alport syndrome, determination of symptomatic and asymptomatic monoallelic *COL4A3*, *COL4A4*, and *COL4A5* carriers (especially *COL4A5* females), and inheritance pattern establishment. Alport syndrome diagnosis confirmation enables clinical course prediction and is crucial for the early introduction of renoprotective treatment with renin–angiotensin–aldosterone system blockade, aimed at slowing the disease progression and estimating the risk in family members, which is important for genetic counselling.

## Data Availability

The original contributions presented in this study are included in the article/Supplementary Materials. Further inquiries can be directed to the corresponding author.

## Supplementary Materials

The following supporting information can be downloaded at https://www.mdpi.com/article/10.3390/genes16020196/s1. Supplementary Table S1: The original Children’s Memorial Health Institute NGS panel of >1000 clinically relevant genes. Supplementary Table S2: Clinical characteristics and genotyping of 247 individuals (138 probands and 109 family members) from 138 families. Supplementary Table S3: Characteristics of 79 molecular variants identified in a group of 109 probands.
